# Distinct requirements for the *C. elegans* Delta ligand APX-1 in embryonic viability and adult fertility

**DOI:** 10.1093/g3journal/jkaf229

**Published:** 2025-09-26

**Authors:** Chien-Hui Chuang, Erin Z Aprison, Ilya Ruvinsky, Bruce Bowerman

**Affiliations:** Institute of Molecular Biology, University of Oregon, Eugene, OR 97403, United States; Department of Molecular Biosciences, Northwestern University, Evanston, IL 60208, United States; Department of Molecular Biosciences, Northwestern University, Evanston, IL 60208, United States; Institute of Molecular Biology, University of Oregon, Eugene, OR 97403, United States

**Keywords:** Notch, Delta, signaling, embryogenesis, fertility, ovulation, vulva development, haplo-insufficiency

## Abstract

Requirements for the *Caenorhabditis elegans* Notch ligand APX-1 have been described, but the molecular lesions in mutant alleles remain unknown. Here we report the sequence changes in 3 previously isolated nonconditional alleles and 2 newly isolated alleles, *apx-1(or545ts)* and a null allele *apx-1(or2015)*. All alleles resulted in highly penetrant embryonic lethality, but only null mutations greatly reduced brood sizes. This reproductive phenotype was likely due to abnormal ovulation rupturing oocytes and, together with vulva defects, resulting in debris accumulation that prevented embryo passage. In addition to identifying molecular lesions in *apx-1* alleles and clarifying distinct requirements for *C. elegans* Notch ligands, our results reveal extensive genotype-by-environment interactions, including haplo-insufficiency of essential loci at a stressfully high growth temperature, and highlight the origins of complex phenotypes as a consequence of multiple seemingly unrelated defects.

## Introduction

The conserved Delta/Notch signaling pathway controls a plethora of animal cell fate decisions. In *Caenorhabditis elegans*, Notch signaling acts through 2 Notch-like receptors, GLP-1 and LIN-12, and 10 Delta-like ligands including APX-1 and LAG-2 (Priess 2005; Greenwald 2013). While the APX-1 and LAG-2 ligands are largely interchangeable, as are both receptors (Fitzgerald et al. 1993; Fitzgerald and Greenwald 1995; Gao and Kimble 1995), they also have somewhat distinct functions. For example, both APX-1 and LAG-2 are expressed in the distal tip cell (Henderson et al. 1994; Gao and Kimble 1995; McGovern et al. 2009; Nadarajan et al. 2009), but only LAG-2 and not APX-1 is strictly required for fertility (Hubbard and Schedl 2019).

Here we focus on APX-1. This Notch ligand mediates an early embryonic induction to specify the fate of a 4-cell stage blastomere (Bowerman et al. 1992; Mango et al. 1994; Mello et al. 1994). Postembryonically, APX-1 functions redundantly with other Delta ligands to mediate both lateral and inductive interactions during vulval and vulval muscle development (Chen and Greenwald 2004; Foehr and Liu 2008) and to sustain ectopic proliferation in tumorous germline mutants (McGovern et al. 2009). Additionally, APX-1 has been reported to be involved in ovulation (McGovern et al. 2018) and to act redundantly with LAG-2 to increase the number of germline progenitor cells (GPCs) (Nadarajan et al. 2009).

Determining APX-1 requirements has been limited by a lack of information as to which mutant alleles most fully reduce gene function. Six recessive *apx-1* alleles have been reported: *zu183*, *zu212*, *zu215*, *or3*, *or15*, and *or22* (Mango et al. 1994; Mello et al. 1994). The *zu183* transposon-insertion allele was used to identify *apx-1* as the disrupted locus; *or3* mapped to near *apx-1* and failed to complement *zu183*, *or15*, and *or22*. The *zu183* allele was shown to be strictly maternal, and the *zu183*, *zu212*, *zu215*, and *or3* alleles were all reported to cause recessive and fully penetrant maternal-effect embryonic lethality (Mango et al. 1994; Mello et al. 1994). The *zu212* allele was shown to be an intron deletion, and the *zu183* allele a 3′UTR Tc1 transposon insertion. However, the molecular lesions within the other alleles have not been reported.

Here, we report an analysis of existing and newly isolated *apx-1* alleles that differentially affect embryonic viability compared to brood size and advance our understanding of the APX-1 roles that promote reproduction.

## Materials and methods

### *C. elegans* strain maintenance and culture

*C. elegans* nematodes were maintained under standard conditions at 20 °C (Brenner 1974). The conditional allele *apx-1(or545ts)* was maintained at 15 °C and shifted to restrictive temperatures (25 °C and 26 °C) as shown in the figures.

### Strains used in this study

N2 was used as the wild-type strain in all experiments. The mutant strains used are in Table 1. The isolation of *or3*, *or15*, and *or22* by chemical mutagenesis was described previously (Mango et al. 1994), and *air-2(or207ts)* and *spd-5(or213ts)* were described previously (Severson et al. 2000; Hamill et al. 2002). The *or545ts* allele was isolated in a screen for temperature-sensitive, embryonic-lethal mutants (Encalada et al. 2000). The *apx-1(tm3438)* deletion allele was provided by the National BioResource Project in Japan; our generation of the *apx-1(or2015)* is described below.

**Table 1.** Strains used in this study.

Strain	Genotype
EU91	apx-1(or3) V/DnT1 (IV;V)
EU148	apx-1(or15) V/DnT1 (IV;V)
EU176	apx-1(or22) V/DnT1 (IV;V)
EU1013	apx-1(or545ts) V
EU3501	apx-1(or2015) V/DnT1 (IV;V)
FX03438	apx-1(tm3438) V/tmC16[tmIs1210]

#### Genotyping

All 8 *apx-1* exons and partial introns of *or3*, *or15*, *or22*, and *or545ts* were commercially sequenced using long-read sequencing (Plasmidsaurus) after PCR amplification and compared to the *apx-1* sequence listed in WormBase (version WS297). The primers used for PCR amplification are in Table 2.

**Table 2.** Primer sequences used for PCR amplification and subsequent DNA sequencing to identify molecular lesions in apx-1 alleles.

Primer	Sequence
apx1_exon_1to3_F	ACGCCTCGCTTTGAAGTCAT
apx1_exon_1to3_R	TTGATCTCTGAGAGTGCCGC
apx1_exon_4to5_F	CGAGCTTGGAAAACGGCAAA
apx1_exon_4to5_R	AATGCCCACTTTCACCGCTA
apx1_exon_6to7_F	GCACCGCGTGGAATTTCTG
apx1_exon_6to7_R	TTCCGGGTGGGTAACACATT
apx1_exon_7to8_F	ATCCCCCTAACCCCTCTCTC
apx1_exon_7to8_R	TGGAGTGGTGAAATTGATGGC

#### CRISPR

To generate the deletion allele *apx-1(or2015)*, crRNAs and PAM sites for *apx-1* were selected using the website http://crispor.tefor.net/. The injection mixture of apx-1 crRNAs, repair oligo (IDT), co-CRISPR marker *dpy-10* repair oligo (IDT), *dpy-10* crRNA (IDT), trRNA (IDT), and Cas9-NLS nucleases (IDT) were injected into wild-type young adults (Arribere et al. 2014). The F1 progeny of the injected animals were first selected for the roller phenotype and then screened and sequence confirmed by PCR. The strain was outcrossed upon balancing the confirmed allele with the balancer DnT1. The *apx-1* guide RNA sequences and repair oligos for *apx-1* null allele (*or2015*) are in Table 3.

**Table 3.** Primer sequences used for CRISPR generation of apx-1(or2015) null allele.

*apx-1* crRNAs	AGGATCGTGTGCTAGAAGGG and TGAAAAAGATCTGCATTTGG
*apx-1* knockout repair oligo	CATTTTTCAGTTTTCCATTAATTTTCCAGGTTCTTTCACCCGCCCAATGCAGATCTTTTTCAAAAAAATTTTCCCTGTCCC
Sequencing primers	ACGCCTCGCTTTGAAGTCAT and TGGAGTGGTGAAATTGATGGC

### Hatching rate and brood size analyses

10 to 30 L4 worms grown at 15 °C or 20 °C of the specified genotypes were singled onto seeded plates and incubated at the designated temperature (15 °C, 20 °C, 25 °C, or 26 °C). Worms grown at 20 °C, 25 °C, and 26 °C were transferred to new plates every 24 to 28 h. Worms grown at 15 °C were transferred to new plates once eggs were laid on the initial plate and then continued transferring every 24 to 28 h on subsequent days. To score embryonic lethality and brood sizes, plates with laid embryos were maintained at the designated temperature for another 24 h after removing the adults (20 °C, 25 °C, or 26 °C) or 48 h (15 °C), and then the number of hatched larvae and unhatched eggs was counted. To score embryonic lethality, embryos laid during the first 2 d after transfer to the restrictive temperature were scored. For brood size analysis, worms were transferred daily to new plates until no more eggs were laid, and all embryos (hatched and unhatched) were summed at the end.

**Fig. 1.**
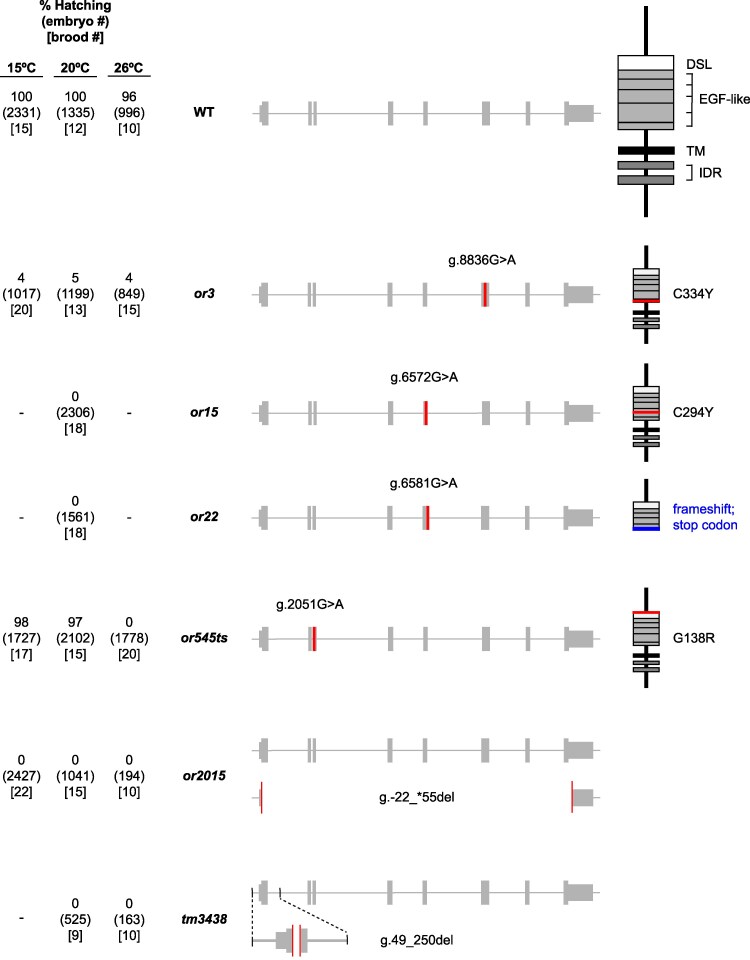
Molecular lesions and embryonic lethality associated with 6 *apx-1* alleles. Schematics of the genomic locus and predicted protein product for 6 loss-of-function *apx-1* mutant loci, showing the locations of nucleotide changes, deletion end points, and amino acid changes. Each mutant locus is annotated with the genomic position(s) of its associated nucleotide change(s), counting from the first nucleotide of the encoded *apx-1* transcript; boxes indicate 5′UTRs, exons, and 3′UTRs. The predicted wild-type protein is annotated to indicate locations of the Delta/Serrate/LAG-2 repeat (DSL), EGF-like repeats, the transmembrane domain (TM), and an intrinsically disordered region (IDR). The % embryonic lethality observed in broods from homozygous hermaphrodites raised at indicated temperatures are to the left, with the total number of embryos in parentheses, and the number of broods that produced those embryos in brackets. See Tables 4 and 5 for alternative depictions of these same data.

**Fig. 2.**
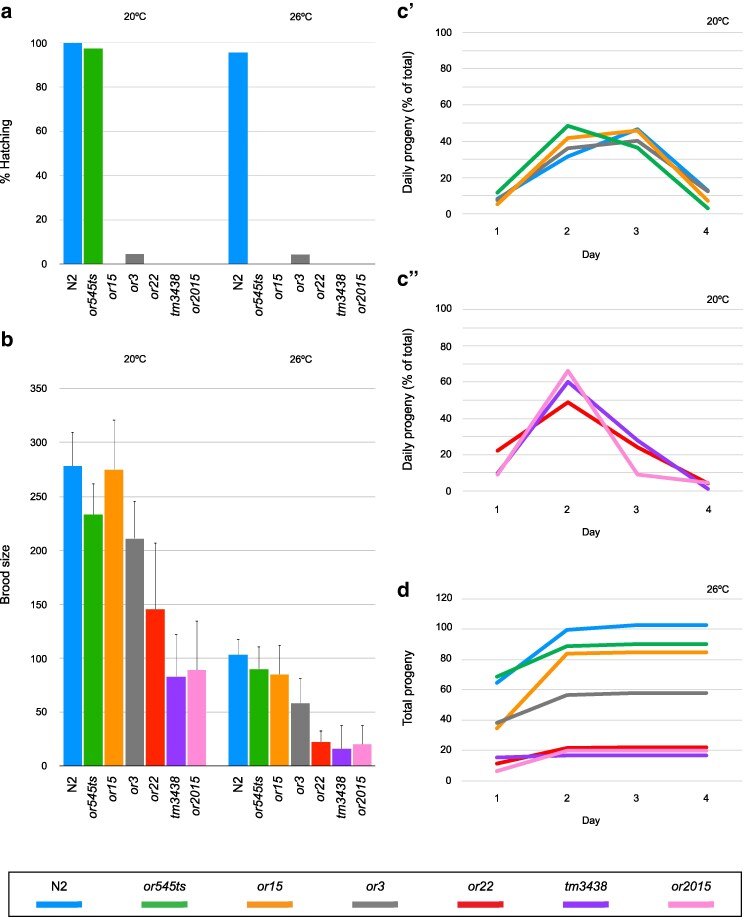
Null alleles of *apx-1* result in a fertility defect. a and b) Bar graphs showing that all 6 *apx-1* mutant alleles resulted in nearly or fully penetrant embryonic lethality a), but only *apx-1* null alleles greatly reduced brood sizes at 20 °C and 26 °C, shown as averages +/− SD. b) The code for mutant alleles at the bottom applies to all panels. c) Number of embryos produced each day: *or3*, *or15*, and *or545ts* mutations resulted in profiles similar to wild type, with broods broadly distributed over days 2 and 3 c′), while the splice donor site mutation *or22* and the 2 deletion mutations *or2015* and *tm3438* resulted in embryo production abruptly ceasing after day 2 c′′). d) Cumulative progeny production at 26 °C. See Table 6 and Supplementary Table 1 for alternative depictions of the same data.

### Culturing worms for imaging germline and egg laying

Synchronized populations were produced by hypochlorite treatment of adult hermaphrodites. Embryos released by hypochlorite treatment (Sulston and Hodgkin 1988) were allowed to hatch in M9 buffer at 15 °C for 24 h for *apx-1(or545ts)* or at 20 °C for 16 h for *apx-1(or2015).* Hatched worms were pipetted onto prepared plates seeded with OP50, and times are measured in hours after this release from L1 arrest. All experiments used N2 wild type, treated in the same manner, as a paired control. We observed no difference in time to adulthood between the *apx-1* mutants and wild type. *apx-1(or2015)* adult hermaphrodites were identified in population plates by their wild-type appearance and their inability to move backward after being touched on the nose.

**Fig. 3.**
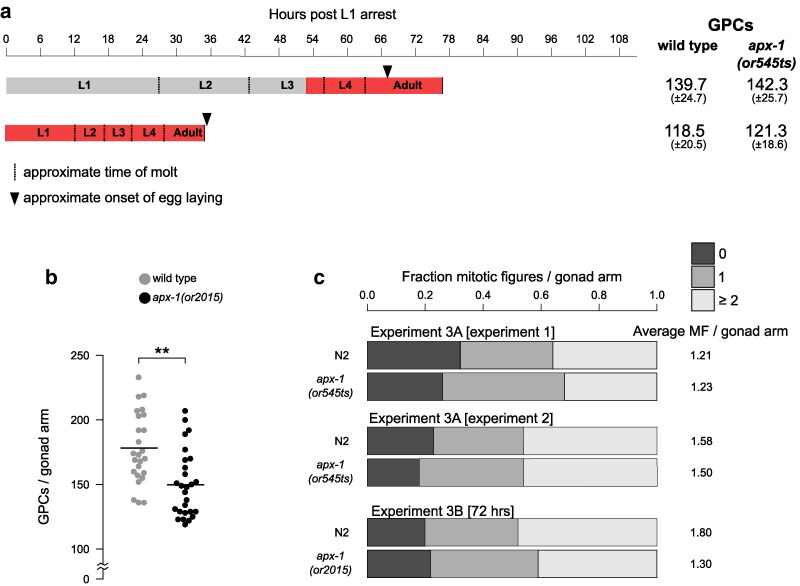
Effects of APX-1 on the GPC population. a) Shifting *apx-1(or545ts)* hermaphrodites from permissive (15 °C, gray) to restrictive (25 °C, red) temperature did not reduce the number of GPCs. Total time from the release from the L1 arrest is shown. Results are given as average ± SD. In all experiments, comparison is with wild type raised in parallel. In all experiments, *N* > 25 for each strain. b) The number of GPCs in wild type and *apx-1(or2015)* hermaphrodites raised in parallel. Each dot represents the number of GPCs per gonad arm in 1 individual. *N* > 25 for each strain; **P < 0.01 Kolmogorov–Smirnov test. Means are indicated by horizontal lines. c) Average numbers and relative fractions of 0, 1, and 2+ mitotic figures (MFs) per gonad arm in the indicated *apx-1* mutant alleles and paired controls.

**Fig. 4.**
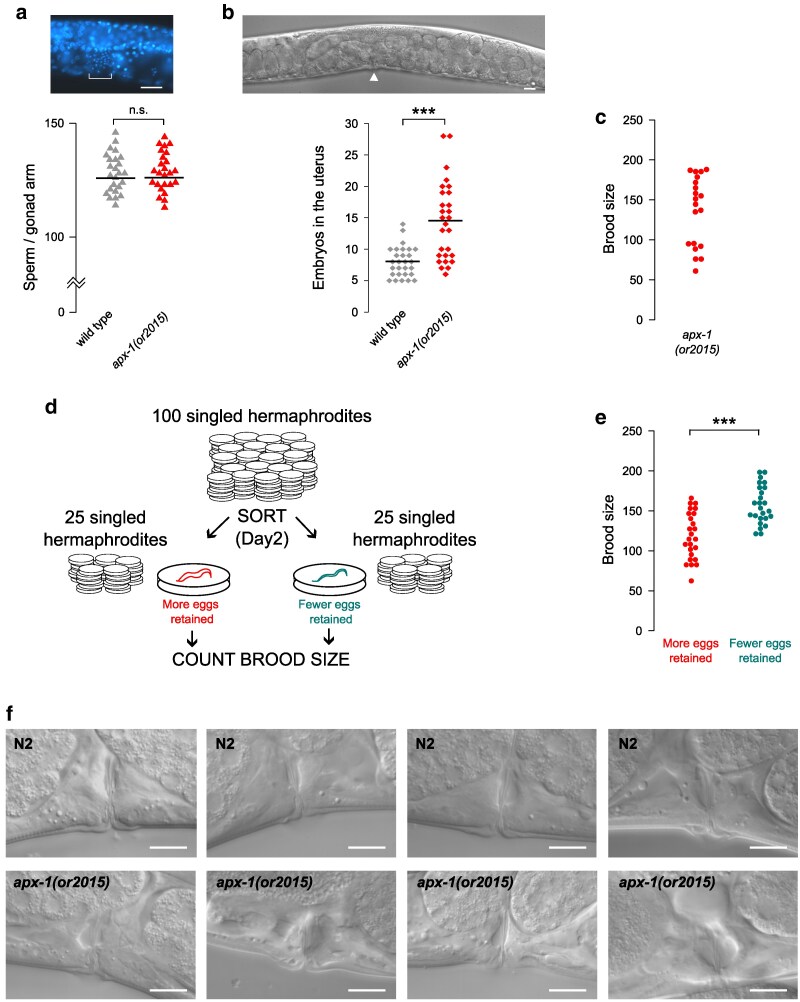
Increased embryo retention in *apx-1* mutants due to vulva defects. a) A representative image of a DAPI-stained young adult hermaphrodite and comparison of the number of sperm cells per gonad arm between *apx-1(or2015)* and wild type. Each triangle represents an individual count (*N* = 25). Location of sperm cells is indicated with a bracket. b) Representative image and quantification of embryos retained in the uterus of *apx-1(or2015)*. The location of the vulva is indicated with an arrowhead. c) The distribution of individual brood sizes appears bimodal in *apx-1(or2015)* hermaphrodites. d) Schematic of the experiment to determine the relationship between the number of embryos retained in the uterus and the subsequent brood size. e) A comparison of brood sizes of *apx-1(or2015)* hermaphrodites with few vs many retained embryos. f) Representative images of vulva morphology in young adult N2 and *apx-1(or2015)* hermaphrodites. See Supplementary Fig. 2 for additional images. In a and b), scale bars are 20 μm, in f) scale bars are 10 μm. ***P < 0.001.

### Counting GPCs and mitotic figures

At the specified times, hermaphrodites were picked into 100 μL phosphate-buffered saline (PBS)-0.1% Tween-20 containing 0.25 mM levamisole. A scalpel was used to cut the worms and extrude the gonads. The dissected worms were washed in M9 and fixed in 95% ethanol for 10 min. Following fixation, the worms were incubated in a Vectashield mounting medium with DAPI (4′,6-diamidino-2-phenylindole) (Vector Laboratories, Burlingame, CA) overnight and mounted on 2% agarose pads. A series of images were taken through the gonad ∼1.5 μm apart on a Leica DM5000B microscope. We determined the boundary of the progenitor zone by identifying crescent-shaped cells (Crittenden et al. 2023) and counted the number of nuclei from the distal tip to the end of the progenitor zone using the “Multi-point” tool in ImageJ. We used the same DAPI preparation of worms to count the number of mitotic figures (Gartner et al. 2004) in the progenitor zone. In each experiment, *apx-1* mutants were processed in parallel with a paired wild-type control. The identity of the gonad—whether *apx-1* mutant or wild type—was blinded to the scorer. Both the GPC counts and mitotic figure numbers are reported as per gonad arm.

### Counting sperm

Wild-type and *apx-1(or2015)* hermaphrodites were DAPI stained as above at 52 h post release from L1 arrest. At this time, some hermaphrodites have completed their first ovulation (Aprison et al. 2022a). This spreads out the sperm and makes them easier to count. Sperm counts are reported as per gonad arm.

### Counting embryos in the uterus

We counted embryos in the uterus in day 2 adult hermaphrodites. As described previously (Aprison et al. 2022b), 20 μL of hypochlorite solution was pipetted into domed PCR caps, and individual hermaphrodites were picked into the solution and incubated for 5 min. We counted the number of hypochlorite-resistant fertilized embryos using a Leica MZ95 stereomicroscope.

### Counting concretions in the uterus

Each day of adulthood, 50 hermaphrodites were anesthetized in 1.25 mM levamisole on 2% agarose pads on 2 glass slides (25 worms/slide). The hermaphrodites were scored for the presence of large masses in the uterus.

### Observing ovulation defects

On day 2 of adulthood, apx-1(or2015) hermaphrodites were anesthetized using a solution of M9 with 0.1% tricaine and 0.01% tetramisole, which does not affect ovulation mechanics (McCarter et al. 1999), and placed on 2% agarose pads.

### Brood size and mating

A synchronized population of *apx-1(or2015)* hermaphrodites was produced by hypochlorite treatment as above. We singled 20 *apx-1(or2015)* hermaphrodites just after the L4/young adult molt. We transferred each hermaphrodite to a fresh plate daily and counted the number of fertilized embryos that had been laid. None of the embryos hatched. On day 3 of adulthood, an additional 20 hermaphrodites were singled from the same population preparation. These hermaphrodites were allowed to mate with 3 day 1 adult N2 males at 15 °C for 2 h. After 2 h, the hermaphrodites were singled to fresh plates. The daily embryo production for these animals was counted as above. None of these embryos hatched.

### Measuring embryo size

On day 2 of adulthood, hermaphrodites were anesthetized with levamisole as above. Fertilized embryos that were released from the hermaphrodites during this treatment were imaged on a Leica DM500B microscope using a Retiga 2000R camera. We used ImageJ to measure the width and length of the embryos. Volume was estimated using the formula: *V* = π *L W*^2^/6, where *L* is the length and *W* is the width of the embryo.

### Data analysis

**Fig. 5.**
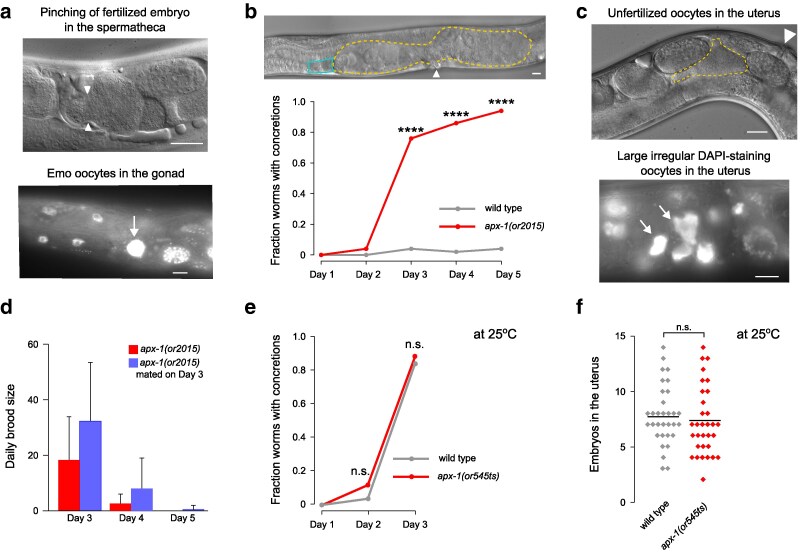
Large masses in the reproductive tracts of *apx-1* mutants. a) Day 2 adult *apx-1(or2015)* hermaphrodites suffer from destructive pinching (arrowheads in the top image) and endomitotic oocytes (Emo; arrow in the bottom image). The image at the top is notably similar to those in Fig. 1 of McGovern et al. (2018), while the bottom image is similar to Fig. 3c of Iwasaki et al. (1996). b) Increase in the incidence of uterine concretions over time. In the *apx-1(or2015)* image, dashed line surrounds the uterus that contains a disorganized mass of yolk, embryos, and debris. A distorted spermatheca is outlined in teal. In a to c), scale bars are 20 μm, ****P < 0.0001. c) Concretions in the uteri of day 2 adult *apx-1(or2015)* hermaphrodites contain unfertilized oocytes (top image) and remnants of endomitotic oocytes (arrows in the bottom image). Arrowhead in the top image marks the vulva. d) Mating on day 3 of adulthood only marginally increases the number of offspring of *apx-1(or2015)* hermaphrodites. *apx-1(or545ts)* hermaphrodites do not e) acquire more uterine concretions or f) retain more embryos than wild type, even at nonpermissive temperature. The substantial difference in the rate of formation of uterine concretions between b and e) is due to the latter reporting experiments at 25 °C, while the former at 20 °C.

All experiments were performed with paired wild-type controls. Statistical significance was evaluated using the Kolmogorov–Smirnov test (Figs. 3b, 4a, b, e, and 5f; Supplementary Fig. 3) or binomial test (Fig. 5b and e).

## Results and discussion

### Conditional, nonconditional, and null *apx-1* alleles

To improve our understanding of APX-1, we analyzed a collection of 6 *apx-1* mutant alleles. These included the 3 nonconditional and recessive alleles *or3*, *or15*, and *or22*, which were isolated previously after chemical mutagenesis (Mango et al. 1994). The molecular nature of the lesions in these 3 alleles has not been reported. We also identified a new temperature-sensitive allele, *or545ts*, which was isolated in a chemical mutagenesis screen for temperature-sensitive, embryonic-lethal mutants, as described (Encalada et al. 2000). We considered *or545ts* to be a candidate *apx-1* allele based on the apparent presence of extra anterior pharyngeal cells in terminally differentiated embryos, as judged by visual inspection using differential interference contrast microscopy. The *or545ts* mutation mapped to the same balancer as *apx-1* and failed to complement *apx-1(or3)* in genetic crosses. All 8 *apx-1* exons and partial introns of *or3*, *or15*, *or22*, and *or545ts* were commercially sequenced using long-read sequencing after PCR amplification and compared to the *apx-1* sequence in WormBase. We thereby identified within these mutant alleles 3 single mis-sense mutations and a splice donor site mutation that may not fully eliminate gene function (Fig. 1; Materials and methods). We therefore used CRISPR/Cas9 genome editing to create *or2015*, a deletion that removes the entire *apx-1* open reading frame (Fig. 1; Materials and methods). We also obtained *tm3438*, a smaller deletion that removes part of the first exon, resulting in a frameshift and premature stop codons. At the standard growth condition of 20 °C, 4 of the nonconditional alleles caused recessive and completely penetrant maternal-effect embryonic lethality, as did *or545ts* at restrictive temperatures, while the penetrance of embryonic lethality for the nonconditional allele *or3* was slightly lower at all tested temperatures (Fig. 1; Tables 4 and 5).

**Table 4.** Temperature sensitivity and haplo-insufficiency of apx-1(or545ts).

Genotype	Temperature (°C)	% Hatching	# Embryos (# broods) scored
			
N2	15	99.66	2331 (15)
N2	20	99.85	1335 (12)
N2	25	98.74	1898 (12)
N2	26	95.58	996 (10)
			
*apx-1(or545ts)*	15	98.09	1727 (17)
*apx-1(or545ts)*	20	97.38	2102 (15)
*apx-1(or545ts)*	25	0.92	1950 (12)
*apx-1(or545ts)*	26	0.07	1778 (20)
*or545ts/+*	15	99.86	1405 (20)
*or545ts/+*	25	99.29	1823 (19)
*or545ts/+*	26	86.70^a^	654 (10)
			
*or2015/+*	25	99.31	726 (12)
*or2015/+*	26	75.23^b^	662 (10)
			
*air-2(or207)/+*	25	99.44	2303 (15)
*air-2(or207)/+*	26	83.76	1127 (14)
			
*spd-5(or213)/+*	25	83.50	1630 (10)
*spd-5(or213)/+*	26	4.63	712 (10)

See Materials and methods for the procedure used to quantify embryonic lethality. Note that for *spd-5(or213ts)/+* at 25 °C, we observed only about 10% embryonic lethality during the first 24 h of brood production (Supplementary Table 1), consistent with the penetrance reported previously (Hamill et al. 2002).

^a^Twenty-five of 110 hatched larvae from *or545ts/+* hermaphrodites, produced after L4 upshifts to 26 °C, made all dead embryos, with the remaining 85 producing viable progeny, indicating that approximately one-fourth (23%) were *or545ts/or545ts*.

^b^Forty-five of 200 hatched larvae from *or2015/+* hermaphrodites, produced after L4 upshifts to 26 °C, made all dead embryos upon maturing to adulthood, with the remaining 155 producing viable progeny, indicating that approximately one-fourth (22.5%) were *or2015/or2015*.

**Table 5.** apx-1 alleles ranked by penetrance of embryonic lethality.

*apx-1* genotype	Temperature (°C)	% Hatching	# Embryos (# broods) scored
			
			
*or2015*	20	0	1041 (15)
*or2015/+*	20	99.65	284 (11)
			
*tm3438*	20	0	525 (9)
*tm3438/+*	20	98.98	782 (7)
			
*or15*	20	0	2306 (18)
*or15/+*	20	99.75	1579 (16)
			
*or22*	20	0	1561 (18)
*or22/+*	20	99.61	1591 (15)
			
*or545ts*	25	0.92	1950 (12)
*or545ts*	26	0.07	1778 (20)
			
*or3*	15	4.03	1017 (20)
*or3*	20	4.59	1199 (13)
*or3*	26	4.48	849 (15)
*or3/+*	15	99.74	1143 (18)
*or3/+*	20	99.73	2225 (16)
*or3/+*	26	94.94	652 (14)

See Materials and methods for the procedure used to score embryonic lethality.

### Haplo-insufficiency of essential loci at 26 °C

When we tested whether *apx-1(or545ts)* is recessive, we were surprised to find evidence for haplo-insufficiency associated with growth at an unusually high temperature. After shifting heterozygous *or545ts/+* hermaphrodites from the permissive (15 °C) to the restrictive (26 °C) temperature during the last (L4) larval stage, 15% of their self-fertilized embryos failed to hatch (Table 4). This embryonic lethality could be due to essential zygotic requirements for *apx-1* during embryogenesis, maternal haplo-insufficiency, or partial maternal dominance (in which an altered mutant protein interferes with wild-type APX-1 function). Approximately one-quarter of the surviving progeny from *or545ts/+* hermaphrodites matured into adults that made dead embryos and were therefore presumably homozygous for *or545ts* (Table 4). We therefore concluded that the embryonic lethality is not due to essential zygotic requirements (or less than one-quarter of the surviving progeny would have produced dead embryos), but rather it is due to maternal haplo-insufficiency or partial maternal dominance.

We next tested whether *apx-1(or545ts)/*+ embryonic lethality at 26 °C might be due to stress associated with growth at this unusually high temperature. Because *C. elegans* strains become sterile at 27.5 °C (Petrella 2014), the restrictive temperature in most screens for heat-sensitive mutants has been 25 °C. However, nearly all wild-type embryos produced at both 25 °C and 26 °C hatch (Table 4), and when isolating *or545ts*, 26 °C was used to maximize the chances of finding heat-sensitive mutations (Encalada et al. 2000). When *or545*ts*/+* L4 hermaphrodites were upshifted to the less stressful temperature of 25 °C, nearly all embryos hatched (Table 4), consistent with *or545ts* being recessive. Furthermore, nearly all embryos hatched after upshifting hermaphrodites heterozygous for the *apx-1* null allele *or2015* from 15 °C to 25 °C, but ∼25% of the embryos died after upshifts to 26 °C, and again roughly one-quarter of the surviving larvae matured into adults that produced all dead embryos (Table 4). The embryonic lethality caused by a null allele ruled out partial maternal dominance. We concluded that the embryonic lethality from heterozygous *apx-1* mutants at 26 °C is a consequence of maternal haplo-insufficiency that results from stress associated with growth at this higher temperature.

We next tested whether temperature-sensitive, recessive mutations in 2 other essential loci that also were isolated at 26 °C, *air-2(or207ts)* and *spd-5(or213ts)*, exhibit maternal haplo-insufficiency due to heat stress. AIR-2 is an Aurora B kinase family member required for chromosome segregation and cytokinesis (Severson et al. 2000), and SPD-5 an essential centrosome scaffolding protein (Hamill et al. 2002). We found that *air-2(or207ts)/+* hermaphrodites produced all viable embryos after upshifts to 25 °C, but over 16% failed to hatch after upshifts to 26 °C (Table 4). The *spd-5(or213ts)* mutation was originally deemed recessive in part because *spd-5* RNAi knockdown resulted in the same centrosome maturation defect observed in *or213ts* mutants (Hamill et al. 2002 ). Nevertheless, in the 48 h following L4 temperature upshifts to 25 °C, we observed nearly 20% embryonic lethality in *spd-5(or213ts)/+* broods (Table 4). Remarkably, we observed almost fully penetrant lethality when *spd-5(or213ts)/+* L4s were upshifted to 26 °C (Table 4). No other *spd-5(-)* alleles have been isolated, and *spd-5* null alleles could be haplo-insufficient even at lower growth temperatures. We concluded that stress associated with growth at 26 °C can result in haplo-insufficiency for essential *C. elegans* loci.

### Distinct effects of *apx-1* alleles on maternal-effect embryonic lethality vs brood size

All *apx-1* alleles were recessive and, except for *or545ts*, caused complete or nearly complete maternal-effect embryonic lethality at both 20 °C and 26 °C (Fig. 2a; Tables 4 and 5). However, only some alleles also resulted in substantially reduced self-progeny brood sizes (Fig. 2b; Table 6). At 20 °C, hermaphrodites homozygous for mis-sense *apx-1* mutations produced nearly wild-type brood sizes, while the 2 deletion alleles *tm3438* and *or2015* both caused nearly 3-fold reductions, and the splice donor site mutation *or22* resulted in an intermediate reduction (Fig. 2b). At 26 °C, *or545ts* and *or15* continued to show nearly wild-type offspring production, while the brood sizes of *or22*, *or2015*, and *tm3438* were severely reduced. The more severe reduction in brood size for *or22* at 26 °C, compared to 20 °C, is consistent with the putative *or22* splicing defect becoming more severe at higher temperatures. Daily offspring production schedules confirmed that reproduction was most severely affected in *or22* and the 2 null alleles *tm3438* and *or2015*, as these homozygous mutants had few if any progeny after day 2 of adulthood when cultured at either 20 °C or 26 °C (Fig. 2c and d; Supplementary Table 1). Finally, the differences in the impacts of *apx-1* alleles on embryonic lethality vs brood size suggest distinct requirements for Notch signaling during embryonic cell fate patterning compared to postembryonic reproductive function—the *or545ts* G138R DSL repeat mutation and the *or15* C294Y EGF-like repeat mutation both eliminate embryonic viability while having little effect on brood size.

**Table 6.** apx-1 alleles ranked by penetrance of brood size reduction.

*apx-1* Genotype	Temperature (°C)	Brood size	# Broods scored
			
N2	15	254 ± 27	15
N2	20	278 ± 32	12
N2	25	189 ± 28	10
N2	26	103 ± 15	10
			
*or2015*	15	116 ± 48	22
*or2015*	20	89 ± 46	30
*or2015*	25	66 ± 43	13
*or2015*	26	20 ± 18	10
			
*tm3438*	20	83 ± 39	9
*tm3438*	26	16 ± 22	10
			
*or3*	15	178 ± 48	20
*or3*	20	211 ± 35	13
*or3*	26	58 ± 23	15
			
*or545ts*	15	258 ± 37	17
*or545ts*	20	233 ± 29	15
*or545ts*	26	90 ± 21	20
			
*or22*	20	145 ± 62	15
*or22*	26	22 ± 11	10
			
*or15*	20	275 ± 46	18
*or15*	26	85 ± 27	20

See Materials and methods for procedure used to score brood size.

### Expansion and maintenance of germline progenitors appear normal in *apx-1* mutants

We next explored possible causes of brood size reduction in *apx-1* null mutants. Because APX-1 is expressed in the distal tip cell of the gonad (Gao and Kimble 1995) that signals to promote germline stem cell identity (Kimble and White 1981), we first examined the GPC population. Previous experiments using RNAi and *apx-1(or3)* concluded that if *apx-1* has a role in maintaining germline progenitors, it is largely redundant with its paralog *lag-2* (Nadarajan et al. 2009). However, *or3* has only a modest brood size reduction (Fig. 2). We therefore carried out 3 sets of experiments with the newly isolated alleles to explore *apx-1* function in germline development. First, we found that shifting the conditional allele *apx-1(or545ts)* to the restrictive temperature (25 °C) during L1 or L3 larval stages did not appreciably change the number of germline progenitors in young adults compared to paired wild-type controls (Fig. 3a). Second, we found that the number of germline progenitors in day 2 adult *apx-1(or2015)* null mutants was moderately but significantly lower than in wild type (Fig. 3b). Consistently, while the number of mitotically dividing germline progenitors in *or545ts* closely mirrored the wild type, *or2015* adult hermaphrodites had fewer mitotic events, though not reaching significance (Fig. 3c). These findings are consistent with a prior report that APX-1 functions largely redundantly with LAG-2 (Nadarajan et al. 2009), which by itself is required for maintaining the stem cell fate of germline progenitors (Henderson et al. 1994). We nevertheless doubted that the modest reduction in the GPCs in the *apx-1* null mutants alone could account for the substantial reduction of brood size.

### APX-1 is required for proper vulva morphology and egg laying

We next examined other possible causes of the brood size reduction in *apx-1* null mutants. A near cessation of egg laying after day 2 of adulthood by worms carrying null alleles (Fig. 2c) could be due to a smaller cache of sperm. However, because the number of sperm in young *apx-1(or2015)* hermaphrodites was approximately the same as in wild type (Fig. 4a), we considered spermatogenesis defects as a less likely explanation of the reduced brood size.

In the course of this work, we noticed that after 1 d of egg laying, *apx-1(or2015)* mutants on average retained approximately twice as many embryos within their uteri as did wild-type hermaphrodites (Fig. 4b). Moreover, these data hinted at the bimodality of the mutant phenotype—while some *apx-1(or2015)* individuals retained as few embryos as the wild type, others retained considerably more. Similarly, the brood size counts also appeared to be bimodally distributed (Fig. 4c; Supplementary Fig. 1). We therefore wondered whether increased embryo retention in some individuals affected the number of offspring they eventually produced. To test this idea directly, from 100 synchronized, singled worms, we selected on day 2 of adulthood 25 that retained many embryos and 25 that, similar to wild type, retained only a few embryos (Fig. 4d). Having counted total progeny production following this selection, we found that the hermaphrodites that had many retained embryos on day 2 generated significantly fewer offspring (Fig. 4e). These results suggest that a defect in egg laying, which would plausibly explain increased embryo retention, may be associated with the reduced reproductive output observed for some *apx-1* mutants.

A requirement for proper egg laying is consistent with previous reports that APX-1 acts redundantly with other Notch ligands to mediate lateral signaling among vulval precursor cells (Chen and Greenwald 2004) and to mediate an inductive interaction that patterns vulval muscle cell fates (Foehr and Liu 2008). We therefore examined vulva morphology in *apx-1(or2015)* during L4 and young adulthood. While we did not observe any obvious differences in vulva morphogenesis during the L4 larval stage, we did detect asymmetries and abnormal morphology of the vulva and vulval muscle cells in young adults (Fig. 4f; Supplementary Fig. 2). We therefore suggest that APX-1 by itself may be required for proper vulva morphogenesis or vulval muscle cell fate patterning and that these defects may be responsible for the embryo retention observed in *apx-1* null mutants.

### APX-1 mutants have defective ovulation and form large uterine masses

How might increased embryo retention be related to reduced reproductive output? Because *apx-1* is expressed in the proximal gonadal sheath and spermatheca cells (McGovern et al. 2009), we tested whether defects in these structures might contribute to the reduced brood sizes. Specifically, we focused on defects in ovulation described at low penetrance in *apx-1(or3)* mutants (McGovern et al. 2018). Consistent with this previous study, in *apx-1(or2015)* hermaphrodites, we observed instances of pinched oocytes and endomitotic oocytes or Emo (Iwasaki et al. 1996) (Fig. 5a).

The embryos produced by *apx-1(or2015)* hermaphrodites exhibited peculiar shapes that were consistent with defective ovulation. While the distribution of *apx-1(or2015)* embryo width overlapped with that of wild-type embryos, the mutant embryos were slightly (∼7%) but significantly shorter (Supplementary Fig. 3a). Due to a strong negative correlation between width and length in *apx-1(or2015)* but not wild-type embryos (Supplementary Fig. 3b), the average embryo volumes between these 2 strains were similar (Supplementary Fig 3c). We inferred that abnormal ovulation in *apx-1(or2015)*, which possibly includes destructive pinching, systematically alters oocyte shape.

Ovulation defects, particularly destructive pinching, may cause maladaptive phenotypes beyond altered embryo shape. We observed that approximately 1 d after embryo retention became evident in *apx-1(or2015)* mutants, these worms accumulated large uterine masses that we call concretions. In their maximal extent, these concretions extended from the somewhat deformed spermatheca through the entire uterus (Fig. 5b). At 20 °C, concretions were never seen in wild-type hermaphrodites, while most *apx-1(or2015)* mutants had them by day 3 and nearly all by day 5. Morphologically, these concretions resembled those that form in uteri of worms that experienced heat shock stress (Aprison and Ruvinsky 2014). In that study, we demonstrated that at higher temperatures, worms experience ovulation defects and form large uterine masses, presumably from mangled embryos and oocytes, which prevent embryo passage. To clarify the composition of uterine concretions in *apx-1(or2015)* hermaphrodites, we examined them in day 2 adults. While the concretions were disorderly and heterogeneous, they contained unfertilized oocytes, which are almost never seen in wild-type animals of this age, and mangled remnants of endomitotic oocytes and embryos (Fig. 5c).

Our results suggest a simple model. Defective ovulation in *apx-1* null mutants yields slightly deformed but fertilizable oocytes and remnants of ruptured oocytes. Vulva defects slow down egg laying to facilitate embryo accumulation in the uterus. These embryos and oocyte remnants form large masses in the uterus, obstructing embryo passage and thus limiting brood size. Two additional lines of evidence support this model. First, when day 3 *apx-1(or2015)* hermaphrodites cultured at 20 °C were mated to young wild-type males (∼75% formed concretions by this time; see Fig. 5b), they produced only slightly more progeny than unmated *apx-1(or2015)* hermaphrodites processed in parallel (Fig. 5d; Supplementary Fig. 4). Because mating typically yields substantially larger broods (Hughes et al. 2007), we inferred that the limited benefit of mating likely was due to either a physical obstruction to sperm migration in the mutant uteri or to the inability of embryos to pass through concretions even when ample sperm were available for fertilization. Second, some *apx-1* alleles showed little if any reduction in self-progeny production, despite causing fully penetrant embryonic lethality (Fig. 2a and b). We examined the reproductive tracts in one such mutant, *apx-1(or545ts)* at the restrictive temperature of 25 °C. Embryo retention and concretion formation were not significantly different from wild type (Fig. 5e and f). Note that at 25 °C, by day 3 of adulthood, concretions were observed in the uteri of most wild-type worms, further limiting their reproductive output. The coincidence of concretions with dramatically reduced fertility observed for *apx-1* null alleles, while both appeared like wild type in *or545ts*, supports a model in which slower egg laying and ovulation defects produce concretions that greatly reduce brood size in *apx-1* null mutants.

### Concluding remarks

We have identified molecular lesions in alleles of the *C. elegans* Notch ligand locus *apx-1*. We found that while all alleles caused highly penetrant embryonic lethality, only those that likely eliminated the gene product reduced brood sizes substantially. We infer that this impairment of reproduction arises from defects in egg laying and ovulation, 2 distinct processes that are both regulated by APX-1. Lastly, we interpret the emergence at higher temperatures of haplo-insufficiency of essential loci, including *apx-1*, as the consequence of stricter requirements for appropriate gene dosage as proteins become less stable during heat stress.

## Supplementary Material

jkaf229_Supplementary_Data

## Data Availability

All strains are available upon request. Supplementary File 1 contains brood sizes for *apx-1(or215)* at specific temperatures. Supplementary File 2 contains DIC images that compare wild-type and *apx-1(or2015)* young adult hermaphrodite vulva morphology. Supplementary File 3 contains a description of wild-type and *apx-1(or2015)* embryo dimensions. Supplementary File 4 contains a graph showing the average daily brood sizes for *apx-1(or2015)*. Table S1 contains daily brood counts for apx-1 alleles at different temperatures. Supplemental material available at *G3* online.

## References

[jkaf229-B1] Aprison EZ, Dzitoyeva S, Angeles-Albores D, Ruvinsky I. 2022a. A male pheromone that improves the quality of the oogenic germline. Proc Natl Acad Sci U S A. 119:e2015576119. 10.1073/pnas.2015576119. 35576466 PMC9173808

[jkaf229-B2] Aprison EZ, Dzitoyeva S, Ruvinsky I. 2022b. The serotonin circuit that coordinates germline proliferation and egg laying with other reproductive functions in *Caenorhabditis elegans*. Proc R Soc B Biol Sci. 289:20220913. 10.1098/rspb.2022.0913. PMC9709507 36448283

[jkaf229-B3] Aprison EZ, Ruvinsky I. 2014. Balanced trade-offs between alternative strategies shape the response of *C. elegans* reproduction to chronic heat stress. PLoS One. 9:e105513. 10.1371/journal.pone.0105513. 25165831 PMC4148340

[jkaf229-B5] Arribere JA et al 2014. Efficient marker-free recovery of custom genetic modifications with CRISPR/Cas9 in *Caenorhabditis elegans*. Genetics. 198:837–846. 10.1534/genetics.114.169730. 25161212 PMC4224173

[jkaf229-B6] Bowerman B, Tax FE, Thomas JH, Priess JR. 1992. Cell interactions involved in development of the bilaterally symmetrical intestinal valve cells during embryogenesis in *Caenorhabditis elegans*. Development. 116:1113–1122. 10.1242/dev.116.4.1113. 1295733

[jkaf229-B7] Brenner S . 1974. The genetics of *caenorhabditis elegans*. Genetics. 77:71–94. 10.1093/genetics/77.1.71. 4366476 PMC1213120

[jkaf229-B8] Chen N, Greenwald I. 2004. The lateral signal for LIN-12/Notch in *C. elegans* vulval development comprises redundant secreted and transmembrane DSL proteins. Dev Cell. 6:183–192. 10.1016/S1534-5807(04)00021-8. 14960273

[jkaf229-B9] Crittenden SL, Seidel HS, Kimble J. 2023. Analysis of the *C. elegans* germline stem cell pool. In: Buszczak M, editors. Germline stem cells, methods in molecular biology. Springer US. p. 1–36. 10.1007/978-1-0716-3259-8_1. 37464233

[jkaf229-B10] Encalada SE et al 2000. DNA replication defects delay cell division and disrupt cell polarity in early *Caenorhabditis elegans* embryos. Dev Biol. 228:225–238. 10.1006/dbio.2000.9965. 11112326

[jkaf229-B11] Fitzgerald K, Greenwald I. 1995. Interchangeability of *Caenorhabditis elegans* DSL proteins and intrinsic signalling activity of their extracellular domains in vivo. Development. 121:4275–4282. 10.1242/dev.121.12.4275. 8575327

[jkaf229-B12] Fitzgerald K, Wilkinson HA, Greenwald I. 1993. *glp-1* can substitute for *lin-12* in specifying cell fate decisions in *Caenorhabditis elegans*. Development. 119:1019–1027. 10.1242/dev.119.4.1019. 8306872

[jkaf229-B13] Foehr ML, Liu J. 2008. Dorsoventral patterning of the *C. elegans* postembryonic mesoderm requires both LIN-12/Notch and TGFβ signaling. Dev Biol. 313:256–266. 10.1016/j.ydbio.2007.10.027. 18036582 PMC2213558

[jkaf229-B14] Gao D, Kimble J. 1995. APX-1 can substitute for its homolog LAG-2 to direct cell interactions throughout *Caenorhabditis elegans* development. Proc Natl Acad Sci U S A. 92:9839–9842. 10.1073/pnas.92.21.9839. 7568229 PMC40898

[jkaf229-B15] Gartner A, MacQueen AJ, Villeneuve AM. 2004. Methods for analyzing checkpoint responses in *Caenorhabditis elegans*. In: Checkpoint controls and Cancer. Humana Press. p. 257–274. 10.1385/1-59259-788-2:257. 15187259

[jkaf229-B16] Greenwald I . 2013. Notch signaling: genetics and structure. WormBook:1–28. 10.1895/wormbook.1.10.2. PMC5402211 23355521

[jkaf229-B17] Hamill DR, Severson AF, Carter JC, Bowerman B. 2002. Centrosome maturation and mitotic spindle assembly in *C. elegans* require SPD-5, a protein with multiple coiled-coil domains. Dev Cell. 3:673–684. 10.1016/S1534-5807(02)00327-1. 12431374

[jkaf229-B18] Henderson ST, Gao D, Lambie EJ, Kimble J. 1994. *lag-2* may encode a signaling ligand for the GLP-1 and LIN-12 receptors of *C. elegans*. Development. 120:2913–2924. 10.1242/dev.120.10.2913. 7607081

[jkaf229-B19] Hubbard EJA, Schedl T. 2019. Biology of the *Caenorhabditis elegans* germline stem cell system. Genetics. 213:1145–1188. 10.1534/genetics.119.300238. 31796552 PMC6893382

[jkaf229-B20] Hughes SE, Evason K, Xiong C, Kornfeld K. 2007. Genetic and pharmacological factors that influence reproductive aging in Nematodes. PLoS Genet. 3:e25. 10.1371/journal.pgen.0030025. 17305431 PMC1797816

[jkaf229-B21] Iwasaki K, McCarter J, Francis R, Schedl T. 1996. emo-1, a *Caenorhabditis elegans* Sec61p gamma homologue, is required for oocyte development and ovulation. J. Cell Biology. 134:699–714. 10.1083/jcb.134.3.699. PMC2120936 8707849

[jkaf229-B22] Kimble JE, White JG. 1981. On the control of germ cell development in *Caenorhabditis elegans*. Dev Biol. 81:208–219. 10.1016/0012-1606(81)90284-0. 7202837

[jkaf229-B23] Mango SE, Thorpe CJ, Martin PR, Chamberlain SH, Bowerman B. 1994. Two maternal genes, *apx-1* and *pie-1*, are required to distinguish the fates of equivalent blastomeres in the early *Caenorhabditis elegans* embryo. Development. 120:2305–2315. 10.1242/dev.120.8.2305. 7925031

[jkaf229-B24] McCarter J, Bartlett B, Dang T, Schedl T. 1999. On the control of oocyte meiotic maturation and ovulation in *Caenorhabditis elegans*. Dev Biol. 205:111–128. 10.1006/dbio.1998.9109. 9882501

[jkaf229-B25] McGovern M et al 2018. The DSL ligand APX-1 is required for normal ovulation in C. elegans. Dev Biol. 435:162–169. 10.1016/j.ydbio.2018.01.009. 29371032 PMC5957500

[jkaf229-B26] McGovern M, Voutev R, Maciejowski J, Corsi AK, Hubbard EJA. 2009. A “latent niche” mechanism for tumor initiation. Proc Natl Acad Sci U S A. 106:11617–11622. 10.1073/pnas.0903768106. 19564624 PMC2710656

[jkaf229-B27] Mello CC, Draper BW, Prless JR. 1994. The maternal genes apx-1 and glp-1 and establishment of dorsal-ventral polarity in the early C. elegans embryo. Cell. 77:95–106. 10.1016/0092-8674(94)90238-0. 8156602

[jkaf229-B28] Nadarajan S, Govindan JA, McGovern M, Hubbard EJA, Greenstein D. 2009. MSP and GLP-1/Notch signaling coordinately regulate actomyosin-dependent cytoplasmic streaming and oocyte growth in *C. elegans*. Development. 136:2223–2234. 10.1242/dev.034603. 19502484 PMC2729341

[jkaf229-B29] Petrella LN . 2014. Natural variants of *C. elegans* demonstrate defects in both sperm function and oogenesis at elevated temperatures. PLoS One. 9:e112377. 10.1371/journal.pone.0112377. 25380048 PMC4224435

[jkaf229-B30] Priess JR . 2005. Notch signaling in the *C. elegans* embryo. WormBook:1–16. 10.1895/wormbook.1.4.1. PMC4780919 18050407

[jkaf229-B31] Severson AF, Hamill DR, Carter JC, Schumacher J, Bowerman B. 2000. The Aurora-related kinase AIR-2 recruits ZEN-4/CeMKLP1 to the mitotic spindle at metaphase and is required for cytokinesis. Curr Biol. 10:1162–1171. 10.1016/S0960-9822(00)00715-6. 11050384

[jkaf229-B32] Sulston J, Hodgkin J. 1988. Methods. In: Wood WB, editors. The nematode Caenorhabditis elegans. Cold Spring Harbor Laboratory, Cold Spring Harbor. p. 587–606.

