# Supplementary material for: Distinct requirements for the C. elegans Delta ligand APX-1 in embryonic viability and adult fertility
Source: G3 (Bethesda). 2025 Sep 26;15(12):jkaf229. doi: 10.1093/g3journal/jkaf229 (PMC12693620; doi:10.1093/g3journal/jkaf229)
Supplement: jkaf229_Supplementary_Data [file jkaf229_supplementary_data.zip › Supplemental_Material_G3-2025-406163.pdf]

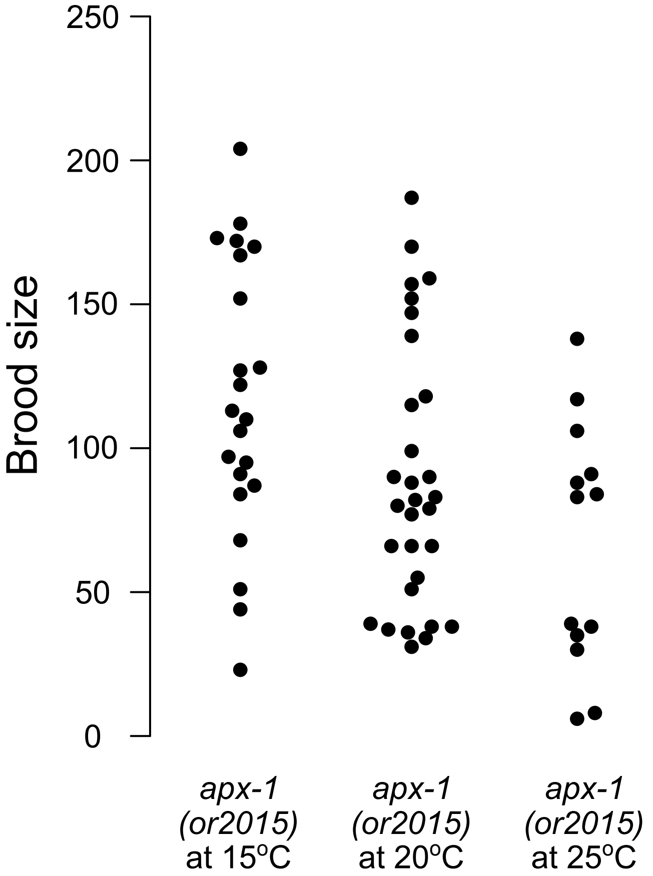

Brood size
250
200
150
100
50
0
apx-1 (or2015) at 15°C
apx-1 (or2015) at 20°C
apx-1 (or2015) at 25°C

Supplemental Figure S1. Self-brood sizes of singled *apx-1(or2015)* hermaphrodites. Each dot represents the number of offspring produced by a single worm. At 20˚C and particularly at 25˚C, average brood sizes are lower. There is also an indication that brood sizes are bimodally distributed, as in Figure 4C. A plausible interpretation of this observation is that at higher temperatures the egg-laying and ovulation defects of *apx-1(or2015)* on average become more pronounced, leading to reduced average brood sizes, while incomplete penetrance results in bimodality of the effect.

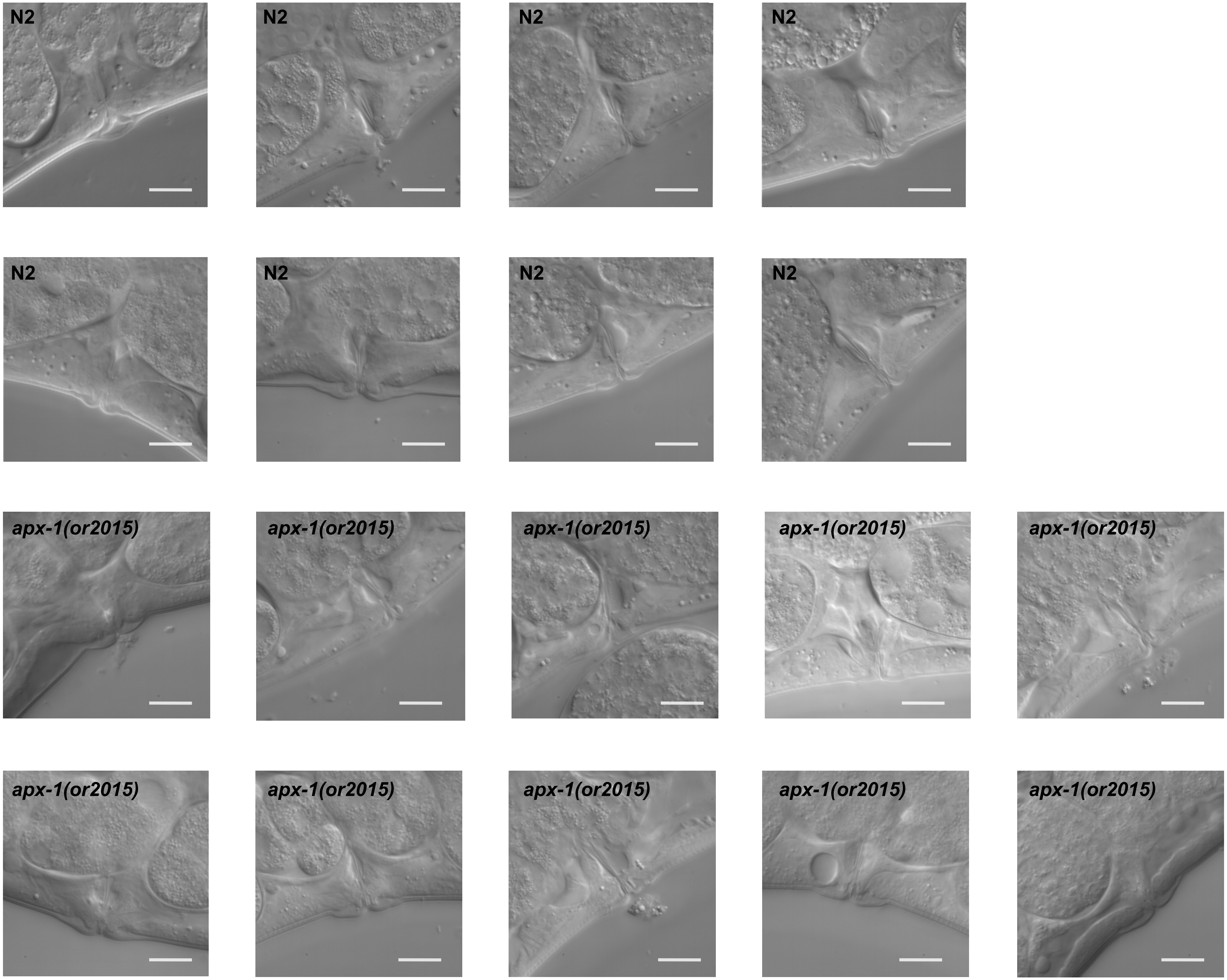
N2
N2
N2
N2
N2
N2
N2
N2
apx-1(or2015)
apx-1(or2015)
apx-1(or2015)
apx-1(or2015)
apx-1(or2015)
apx-1(or2015)
apx-1(or2015)
apx-1(or2015)
apx-1(or2015)
apx-1(or2015)

Supplemental Figure S2. Comparison of vulva morphology of wild type N2 and *apx-1(or2015)* young adult hermaphrodites. Despite individual-to-individual variability, when compared to similarly aged wild type worms, *apx-1(or2015)* hermaphrodites show notably reduced symmetry around the vulva opening and vulva muscles. Scale bars are 10 µm.

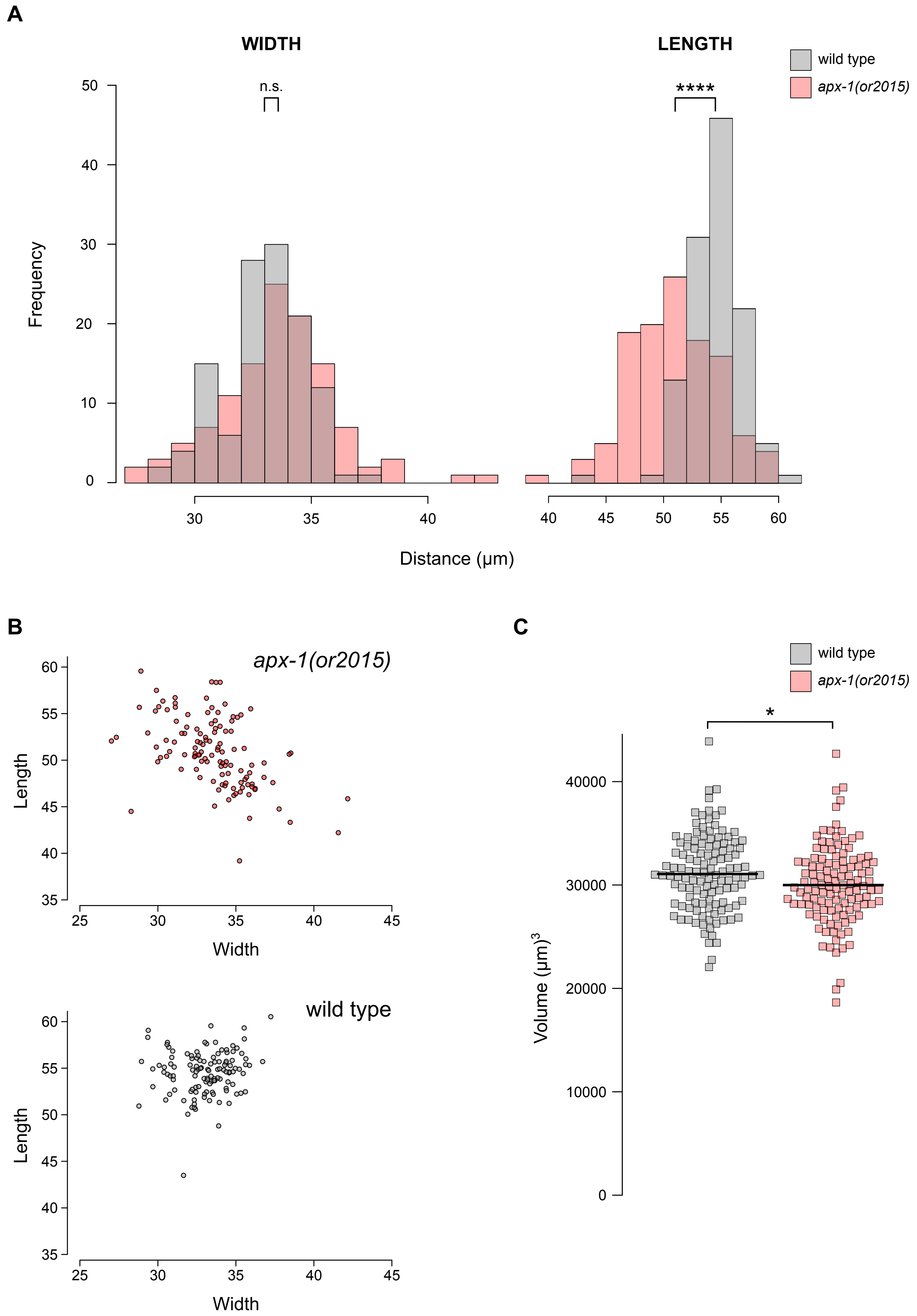

A
WIDTH
LENGTH
wild type
apx-1(or2015)
n.s.
****
Frequency
50
40
30
20
10
0
30
35
40
45
50
55
60
Distance (μm)
B
apx-1(or2015)
Length
Width
25
30
35
40
45
wild type
Length
Width
C
wild type
apx-1(or2015)
*
Volume (μm)3
40000
30000
20000
10000
0

Supplemental Figure S3. Dimensions of *apx-1(or2015)* and wild type embryos. (A) Distributions of width and length of embryos collected on Day 2 of parents' adulthood. The average width of *apx-1(or2015)* embryos is ~2% greater than wildtype, but the average length ~7% lower. ****, $p<0.0001$. Although the data are presented as frequency histograms for clarity, statistical analyses were performed on primary data using the Kolmogorov-Smirnov test. Actual p-value = 8.3^{-15} (B) Negative correlation between width and length in *apx-1(or2015)*, but not wild type embryos. Pearson correlations are -0.5 and 0.14 for *apx-1(or2015)* and wild type embryos, respectively. (C) Distributions of estimated embryo volumes. *, $p<0.05$. The average volume of *apx-1(or2015)* embryos is ~4% smaller than wildtype. The difference is marginally statistically significant using the Kolmogorov-Smirnov test. Actual p-value = 0.0499.

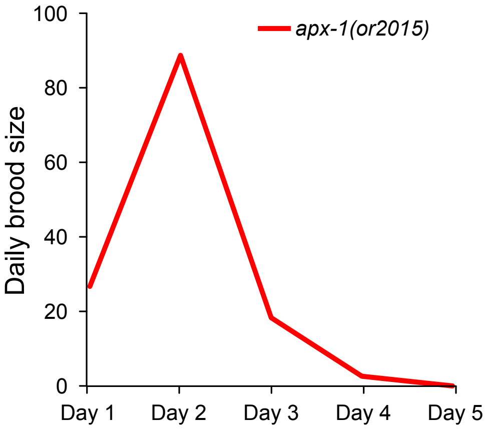
apx-1(or2015)
Daily brood size
100
80
60
40
20
0
Day 1
Day 2
Day 3
Day 4
Day 5

Supplemental Figure S4. Daily progeny production by unmated *apx-1(or2015)* hermaphrodites. The plot shows the average number of self-progeny produced on each day of adulthood. Based on 20 individually-housed hermaphrodites.
