# Supplementary material for: Distinct requirements for the C. elegans Delta ligand APX-1 in embryonic viability and adult fertility
Source: G3 (Bethesda). 2025 Sep 26;15(12):jkaf229. doi: 10.1093/g3journal/jkaf229 (PMC12693620; doi:10.1093/g3journal/jkaf229)
Supplement: jkaf229_Supplementary_Data [file jkaf229_supplementary_data.zip › Table_S1_G3-2025-406163.pdf]

Table S1. Daily brood production counts for each genotype and temperature

**N2**	Day 1	Day 2	Day 3	Day 4	Day 5	Day 6
15C	68.47 ± 12.89	86.93 ± 11.06	78.0 ± 14.83	18.47 ± 14.62	1.87 ± 3.00	0
20C	23.42 ± 10.28	87.83 ± 17.41	130.25 ± 16.28	36.0 ± 19.05	0.17 ± 0.39	0
25C	53.1 ± 8.86	112.4 ± 26.34	21.5 ± 11.45	2.3 ± 3.97	0.1 ± 0.32	0
26C	64.5 ± 12.48	35.1 ± 7.03	3.1 ± 2.64	0	0	-

or545ts	Day 1	Day 2	Day 3	Day 4	Day 5	Day 6	Day7
15C	23.47 ± 8.99	78.12 ± 11.40	95.59 ± 12.30	51.71 ± 25.59	8.0 ± 7.65	1.06 ± 1.09	0
20C	27.13 ± 9.68	113.0 ± 14.59	85.2 ± 29.09	7.2 ± 9.39	0.13 ± 0.52	0	-
25C	57.09 ± 12.45	92.73 ± 33.27	7.82 ± 8.61	0	0	-	-
26C	68.6 ± 16.66	20.3 ± 8.86	1.4 ± 3.02	0.05 ± 0.22	0	-	-

**or3**	Day 1	Day 2	Day 3	Day 4	Day 5	Day 6	Day7
15C	8.8 ± 5.70	42.05 ± 13.56	55.55 ± 15.96	59.05 ± 25.60	10.4 ± 17.81	2.45 ± 7.60	0
20C	16.31 ± 7.64	75.92 ± 18.98	84.77 ± 17.49	25.54 ± 22.60	7.62 ± 20.03	0.77 ± 1.92	0
25C	-	-	-	-	-	-	-
26C	38.0 ± 18.88	18.6 ± 10.05	1.07 ± 2.84	0	-	-	-

**or15**	Day 1	Day 2	Day 3	Day 4	Day 5	Day 6	Day7
15C	-	-	-	-	-	-	-
20C	13.56 ± 6.40	114.56 ± 15.52	125.72 ± 26.33	19.89 ± 16.33	0.61 ± 1.24	0.39 ± 0.92	0
25C	-	-	-	-	-	-	-
26C	34.55 ± 14.83	49.65 ± 20.78	0.7 ± 1.3	0	-	-	-

**or22**	Day 1	Day 2	Day 3	Day 4	Day 5	Day 6	Day7
15C	-	-	-	-	-	-	-
20C	31.6 ± 25.85	71.07 ± 29.58	34.8 ± 26.07	5.73 ± 10.33	0.73 ± 1.49	0.73 ± 2.15	0
25C	-	-	-	-	-	-	-
26C	11.2 ± 6.89	10.3 ± 6.98	0.3 ± 0.67	0	-	-	-

**or2015**	Day 1	Day 2	Day 3	Day 4	Day 5	Day 6
15C	72.41 ± 27.15	37.91 ± 29.17	5.0 ± 9.69	1.14 ± 1.64	0	-
20C	8.03 ± 7.23	59.4 ± 26.29	17.57 ± 18.42	3.87 ± 6.48	0.1 ± 0.31	0
25C	30.15 ± 21.65	35.54 ± 26.68	0.69 ± 1.44	0	-	-
26C	6.0 ± 6.46	13.4 ± 13.68	0.3 ± 0.67	0	-	-

**tm3438**	Day 1	Day 2	Day 3	Day 4	Day 5	Day 6
15C	-	-	-	-	-	-
20C	8.11 ± 9.41	50.22 ± 22.82	23.0 ± 22.30	1.33 ± 1.73	0	-
25C	-	-	-	-	-	-
26C	15.1 ± 20.50	1.2 ± 1.32	0	0	-	-
